# Long-term exposure to air pollution and risk of venous thromboembolism in a large administrative cohort

**DOI:** 10.1186/s12940-022-00834-2

**Published:** 2022-01-27

**Authors:** Matteo Renzi, Massimo Stafoggia, Paola Michelozzi, Marina Davoli, Francesco Forastiere, Angelo G. Solimini

**Affiliations:** 1 Department of Epidemiology, Health Authority Service, ASL Rome 1, 00147 Rome, Italy; 2 Department of Health Statistics and Biometry, University of Rome “La Sapienza”, Rome, Italy; 3 Institute of Environmental Medicine, Karolinska Instituet, Stockholm, Sweden; 4 National Research Council of Italy, Institute of Innovation and Biomedical Research (IRIB), , Palermo, Italy; 5 Department of Public Health and Infectious Diseases, University of Rome “La Sapienza”, Rome, Italy

**Keywords:** Air pollution, Cohort, Deep vein thrombosis, Pulmonary embolism, Venous thromboembolisms

## Abstract

**Background:**

Venous thromboembolisms (VTE) are one of the most frequent cause among the cardiovascular diseases. Despite the association between long-term exposure to air pollution and cardiovascular outcomes have been widely explored in epidemiological literature, little is known about the air pollution related effects on VTE. We aimed to evaluate this association in a large administrative cohort in 15 years of follow-up.

**Methods:**

Air pollution exposure (NO_2_, PM_10_ and PM_2.5_) was derived by land use regression models obtained by the ESCAPE framework. Administrative health databases were used to identify VTE cases. To estimate the association between air pollutant exposures and risk of hospitalizations for VTE (in total and divided in deep vein thrombosis (DVT) and pulmonary embolism (PE)), we used Cox regression models, considering individual, environmental (noise and green areas), and contextual characteristics. Finally, we considered potential effect modification for individual covariates and previous comorbidities.

**Results:**

We identified 1,954 prevalent cases at baseline and 20,304 cases during the follow-up period. We found positive associations between PM_2.5_ exposures and DVT, PE and VTE with hazard ratios (HRs) up to 1.082 (95% confidence intervals: 0.992, 1.181), 1.136 (0.994, 1.298) and 1.074 (0.996, 1.158) respectively for 10 μg/m^3^ increases. The association was stronger in younger subjects (< 70 years old compared to > 70 years old) and among those who had cancer.

**Conclusion:**

The effect of pollutants on PE and VTE hospitalizations, although marginally non-significant, should be interpreted as suggestive of a health effect that deserves attention in future studies.

**Supplementary Information:**

The online version contains supplementary material available at 10.1186/s12940-022-00834-2.

## Essentials:

1. Relationship between VTE and air pollution is not well established in the epidemiological literature
2. Rome longitudinal study (RoLS) is a large administrative cohort composed by more than 1 million of subjects
3. Increases in pollutant concentrations are related to increases in risk of hospitalizations for DVT and PE
4. As the VTE are the third most frequent CVD an important need to increase the available evidence about the risk factor is raised.

## Introduction

Long-term and short-term exposures to air pollutants are associated with an increased morbidity and mortality, especially from cardiovascular (CVD) and respiratory diseases [1–5]. Venous thromboembolisms (VTE) represent the third most frequent causes cardiovascular diseases, including deep vein thrombosis (DVT) and pulmonary embolism (PE). VTE episodes are important cause of cardiovascular morbidity and mortality [6] with a global burden of disease of about 10 million of cases per year [7]. The National Heart, Lung and Blood Institute defines VTE as “a disorder that includes deep vein thrombosis and pulmonary embolism. A deep vein thrombosis (DVT) occurs when a blood clot forms in a deep vein, usually in the lower leg, thigh, or pelvis [8]. A pulmonary embolism (PE) occurs when a clot breaks loose and travels through the bloodstream to the lungs”. It is estimated that PE account for 10 to 30% of fatal cases within 30 days from diagnosis (Beckman 2010). The annual incidence of VTE were estimated to be around 1–2 per 1000 inhabitants in the US (DVT and PE together [9]) and 148 and 95 per 100,000 in the EU (for DVT and PE respectively [6],). Risk of VTE is higher in hospitalized patients, especially those who had received surgery or after trauma procedures, because of venous stasis, immobilization and/or limited postoperative mobility in addition to the activation of inflammatory and coagulation pathways after tissue injury. Up to 60% of all cases of VTE occur within 90 days of hospitalization [10]. Known risk factors include older age, cancer, autoimmune and chronic inflammatory diseases, varicose veins, obesity, inactivity, smoking, estrogen replacement and pregnancy for women.

Exposure to air pollutant mixtures could increase the likelihood of VTE events through multiple and interdependent mechanisms [11]. Particulate matter (PM) is a mixture of solid particles and liquid droplets. Principally, it is classified by the mass median aerodynamic diameter of the particles in PM_10_, composed by less than 10-micron particles, and PM_2.5_, where particles are less than 2.5 micron. PM inhalation activates blood coagulation mechanisms with the production of pro-inflammatory (reactive oxygen species), pro-oxidative proteins (cytokines, C-reactive protein) and hormones (endothelins) and consequent high levels of fibrinogen and thrombin formation [12, 13]. A second mechanism involves the unbalance of the autonomic control of heart rate and consequent decreased heart rate variability through the stimulation of the pulmonary sensory nerves following PM inhalation [14]. Additionally, increased blood pressure and atherosclerotic plaque formation were also reported [15, 16]

Despite plausible biological mechanisms, evidence of air pollution effects on VTE are incomplete and inconclusive. Two cohort studies [17, 18] and one case–control [19] have examined, so far, the association between long-term exposure to air pollutants and VTE but their findings were contrasting and a subsequent meta-analytic analysis of those three studies found an effect estimate close to the null [20]. This study aimed to assess the long-term effect of air pollution (PM_10_, PM_2.5_ and nitrogen dioxide [NO_2_]) on hospitalizations for VTE, DVT and PE in a large administrative cohort in Rome. Additionally, we studied the influence of several effect modifiers, including known risk factors for VTE.

## Material and methods

### Study population

We analyzed data from the Rome Longitudinal Study (ROLS), an administrative cohort based on the 2001 Italian Census operated by the National Institute of Statistics (ISTAT) [21]. We followed more than 1 million of persons from 20–10-2001, the date of the Census, until 31–12-2015. For each subject, we have information about socio-demographic characteristics such as age, sex, place of birth, marital status, educational level, and occupation and residence address. Moreover, we considered also a small-area index assigned to each census block (average population of 470 inhabitants) which is a composite index of socioeconomic position (SEP) [22]. Each subject was identified by an anonymous ID to allow the record linkage with health databases such as the Hospital Registry Discharge (HIS) and the Pharmaceutical databases. Record linkage procedures were anonymous, permitted since the Rome Longitudinal Study is part of the National Statistical Program for the years 2011–2017, and was approved by the Italian Data Protection Authority. More information are available elsewhere [23, 24]. Prevalent VTE cases and subjets who moved outside Rome during the follow-up were excluded at the baseline.

### Outcome definition

Deep vein thrombosis (DVT) and pulmonary embolism (PE) were defined by using the hospitalizations (HIS) database. We used the International Classification of Diseases – 9^th^ version (ICD-9) to classify the different outcomes considering both primary and secondary diagnoses. In particular, the codes considered for DVT definition were: 451–453; while PE is defined by the code 415.1. We excluded the prevalent cases by applying the same definition to each subject in the three years period before the baseline (20–10-2001). VTE cases are defined as a combination of DVT and PE outcomes.

### Exposure assessment

We estimated average annual exposure to air pollutants (PM_10_, PM_2.5_, and NO_2_) at baseline (January 1^st^, 2001) residential address for each individual by land-use regression (LUR) models, developed using measures taken in 2010 within the ESCAPE project [25, 26]. The model displayed good prediction properties, with percent explained variability in hold-out monitors ranging between 76% (NO_2_) and 59% (PM_10_) in Rome [27, 28].

We considered green space and noise in a 1-km^2^ area around the residential address as potential confounders of the association between air pollution and VTE. To characterize green space, we used Normalized Difference Vegetation Index (NDVI) calculated by Landsat 5 Thematic Mapper (TM) satellite images (http:// earthexplorer.usgs.gov/). NDVI is a common indicator of green vegetation and was developed using the analysis of surface reflectance measurements. The NDVI values range from − 1 to + 1, with + 1 indicating a high density of green leaves, − 1 representing water features and values close to zero referring to barren areas of rock, sand or snow [29]. The process is described in details elsewhere [30]. Similarly, we account for residential noise exposure as a confounder. Noise was assessed using annual day-evening-night A-weighted equivalent continuous noise levels (Lden) indicator, defined by the European Environmental Agency [31] as average sound pressure level over all days, evenings and nights in a year. Lden was estimated using an approach described in detail elsewhere [32, 33].

### Statistical analysis

The association between long-term exposure to air pollutants and hospitalizations for VTE, DVT and PE was estimated using Cox proportional hazard regression models with age as time scale. We defined four models a priori: model 1 adjusted for only age and sex. We added individual- and area-level confounders such as place of birth (Rome or other), occupational status (employed, unemployed, housewife, retired and other), marital status (married, single, separated/divorced or widowed), area-based socio-economic position (SEP in five levels: from high to low) and educational level (primary, secondary, high school and university) in model 2. Variables which did not meet the proportionality of hazards assumption were included in model 2 as stratification terms. These included: sex, marital status, and SEP. In model 3, we adjusted also for noise by adding a linear term for Lden to model 2. Finally, in addition to the previous set of covariates (model 3), we included greenness with a linear term for NDVI (model 4).

We evaluated possible effect modification by age (classified in two classes: < 70 and > 70 years), sex, and previous comorbidities (cardiovascular diseases, myocardial infarction, hypertension, COPD, diabetes, hyperlipidemia and cancer) by including suitable interaction terms in model 2. In addition, we considered age divided into three classes by terciles (< 40, >  = 40 and < 65, and > 65 years old) as supplementary analyses (see supplementary material). All effect modifiers are considered as time-fixed covariates or at the baseline (i.e. for age). We calculated P-values for interaction using the Wald test.

We applied exposure–response functions to test the association even at low levels of air pollution. Exposure–response curves were obtained by modelling the exposures with natural splines with 3 degrees of freedom.

Finally, as a sensitivity analysis to take into account possible spatial autocorrelation we computed models (model3) with a cluster term for a spatial indicator. This variable assumes 94 different levels according to the different zone in Rome area. The results are provided in the Supplemental material.

We expressed the results as hazard ratios (HRs) and 95% confidence intervals (95%CI) per 10 µg/m^3^ increments in PM_10_, NO_2_, and PM_2.5_.

All analyses were conducted in R software [34].

## Results

Cohort characteristics are reported in Table 1. The cohort is composed by 1,261,770 eligible subjects at the baseline (20 October 2001) after the exclusion of 1,226 prevalent subjects (0.09%). We removed 14,974 (1.2%) records because of missing information on exposure assessment. We obtained a final cohort of 1,260,544 (98.6%) subjects and a total of 15,190,594 person-years of follow-up. Females were 54.5%, while subjects > 70yrs old at the baseline were 19.5(%). At baseline, 173,651 subjects (13.8%) had past admissions for CVD, 143,389 (11.4%) for cancer, 171,643 (13.6%) for COPD and 150,441 (11.9%) for diabetes (Table 1). We observed an incidence rate of 4 and 9.8 per 10,000 person-years for PE and DVT, respectively.

**Table 1:** Individual characteristics of the study cohort at the baseline and incidence of deep vein thrombosis (DVT) and pulmonary embolism (PE)

Variable	N	%	New DVT cases	New PE cases	P-Y	DVT Incidence rate*10,000	PE Incidence rate*10,000
**Total**	1,260,544	100	14,291	6,013	15,190,594	9.41	3.96
** Sex**							
* Males*	574,177	45.5	6,674	2,649	6,808,369	9.80	3.89
* Females*	686,367	54.5	8,247	3,364	8,382,225	9.84	4.01
**Age**							
< *35*	123,825	9.8	563	117	1,626,949	3.46	0.72
* 35–70*	890,578	70.7	9,094	3,317	11,365,819	8.00	2.92
> *70*	246,141	19.5	5,264	2,579	2,197,826	23.95	11.73
**Socio-economic position (area level)**							
* High*	249,525	19.8	2,421	1,207	3,009,906	8.04	4.01
* Medium–High*	256,930	20.4	2,748	1,220	3,082,163	8.92	3.96
* Medium*	252,930	20.1	2,914	1,218	3,036,834	9.60	4.01
* Medium–Low*	256,883	20.4	3,371	1,243	3,097,478	10.88	4.01
* Low*	244,276	19.4	3,467	1,125	2,964,213	11.70	3.80
**Educational level**							
< = *primary school*	313,258	24.9	6,160	2,506	3,400,437	18.12	7.37
* High-school*	415,459	33.0	3,201	1,314	5,239,336	6.11	2.51
* Junior-school*	326,960	25.9	4,128	1,489	3,976,175	10.38	3.74
* University*	204,867	16.3	1,432	704	2,574,646	5.56	2.73
**Marital status**							
* Married*	836,296	66.3	9,537	3,695	10,320,350	9.24	3.58
* Separated*	88,443	7.0	989	349	1,083,201	9.13	3.22
* Singles*	192,469	15.3	1,502	600	2,390,028	6.28	2.51
* Widows*	143,336	11.4	2,893	1,369	1,391,015	20.80	9.84

The average exposure levels are reported in Table 2. We observed average exposures equals to 36.6 µg/m^3^ (standard deviation = 5.1) for PM_10_, 19.6 (1.9) for PM_2.5_ and 42.8 (10.2) for NO_2_. Average noise (Lden) exposure was 59.8 (8.6) dB, while average greenness index was 0.369 (0.126).

**Table 2:** Environmental data description

Pollutant	U	Mean	SD	Min	Percentiles			Max	IQR
					**25th**	**50th**	**75th**		
PM_10_	µg/m^3^	36.6	5.1	29.6	33.3	35.2	38	58.2	4.7
PM_2.5_	µg/m^3^	19.6	1.9	17	18.4	19.1	20.1	27.4	1.7
NO_2_	µg/m^3^	42.8	10.2	13.2	35.7	42.2	48.6	84.9	12.9
Lden	dB	59.8	8.6	17	53.9	60.1	66	89.7	12.1
NDVI	-	0.369	0.126	-0.3	0.308	0.374	0.448	0.772	0.14

Table 3 shows the results of the different models 1–4 of the association between long-term exposure to air pollutants and hospitalization for DVT, PE and VTE. In model 1, we observed no associations for DVT and VTE, while some positive signals emerged for PE outcomes. When we adjusted for individual and area-level confounders in model 2, we observed positive associations especially for PM_2.5_ with HRs up to 1.082 (95%CI: 0.992, 1.181), 1.136 (0.994, 1.298) and 1.074 (0.996, 1.158) for DVT, PE and VTE respectively. No associations were observed for NO_2_ exposure, except for PE outcome. We observed similar affects after adjusting for noise and greenness in model 3 and 4 (see Supplementary Table 1).

**Table 3:** Associations between long-term exposure to air pollutants (PM_10_, PM_2.5_ and NO_2_) and deep vein thrombosis (DVT) and pulmonary embolism (PE) in RoLS. Results are expressed as hazard ratios (HR) with relative 95% confidence intervals per 10 µg/m^3^ increases of pollutant

	**Model 1**^**a**^			**Model 2**^**b**^			**Model 3**^**c**^			**Model 4**^**d**^		
	**HR**	**95%CI**		**HR**	**95%CI**		**HR**	**95%CI**		**HR**	**95%CI**	
**DVT**												
* PM*_*10*_	1.000	0.969	1.032	1.029	0.996	1.062	1.034	0.997	1.072	1.035	0.998	1.073
* PM*_*2.5*_	0.992	0.912	1.080	1.082	0.992	1.181	1.099	0.995	1.214	1.115	1.009	1.233
* NO*_*2*_	0.997	0.982	1.013	0.996	0.979	1.013	0.992	0.973	1.012	1.000	0.980	1.021
**PE**												
* PM*_*10*_	1.038	0.990	1.089	1.044	0.994	1.096	1.038	0.981	1.098	1.037	0.980	1.097
* PM*_*2.5*_	1.110	0.976	1.264	1.136	0.994	1.298	1.123	0.962	1.310	1.109	0.950	1.296
* NO*_*2*_	1.016	0.992	1.042	1.019	0.992	1.047	1.014	0.983	1.047	1.007	0.974	1.040
**VTE**												
* PM*_*10*_	1.002	0.975	1.030	1.025	0.997	1.054	1.029	0.997	1.061	1.029	0.997	1.062
* PM*_*2.5*_	0.999	0.929	1.075	1.074	0.996	1.158	1.086	0.996	1.184	1.095	1.003	1.194
* NO*_*2*_	1.000	0.986	1.014	0.999	0.984	1.014	0.996	0.979	1.014	1.001	0.983	1.019

^a^Model 1 is adjusted for age and sex

^b^Model 2 is adjusted as model 1 + marital status, place of birth, income, socio-economic position (area index), educational level, occupation, and unemployment rate (area level)

^c^Model 3 is adjusted as model 2 + noise

^d^Model 4 is adjusted as model 3 + greenness

In Table 4, we reported the results for the effect modification by age, sex, and previous comorbidities only for PM_2.5_ exposure. No large differences emerged from the analysis for DVT, PE and PM_2.5_ exposure. However, slightly higher effects were observed for younger subjects (< 70 years), and subjects with cancer and no COPD. When we consider different age classes, the results were very similar to those obtained by using the 70 years as cutoff.

**Table 4:** Effect modification of the associations between long-term exposure to PM_2.5_ and deep vein thrombosis and pulmonary embolism in RoLS. Results are expressed as hazard ratios (HR) with relative 95% confidence intervals per 10 µg/m^3^ increases of pollutant

	**DVT**				**PE**			
	HR	95%^a^CI		*P*-value^**^	HR	95%CI		*P*-value
Overall^a^	1.082	0.992	1.181		1.136	0.994	1.298	
Age class								
< *70 y*	1.109	0.994	1.237	-	1.192	0.998	1.424	-
> *70 y*	1.032	0.899	1.185	0.043	1.081	0.890	1.315	0.837
Sex								
* Females*	1.130	0.995	1.285	-	1.168	0.958	1.425	-
* Males*	1.046	0.933	1.173	0.799	1.112	0.933	1.325	0.772
Cardiovascular								
* No*	1.092	0.994	1.199	-	1.180	1.023	1.361	-
* Yes*	1.028	0.824	1.284	0.792	0.896	0.629	1.275	0.185
AMI								
* No*	1.079	0.986	1.181	-	1.117	0.972	1.283	-
* Yes*	1.130	0.810	1.577	0.459	1.407	0.882	2.245	0.901
Hypertension								
* No*	1.079	0.986	1.182	-	1.175	1.023	1.349	-
* Yes*	1.112	0.834	1.481	0.777	0.777	0.487	1.240	0.071
Diabetes								
* No*	1.084	0.984	1.193	-	1.156	0.999	1.338	-
* Yes*	1.078	0.887	1.310	0.554	1.044	0.766	1.423	0.539
Hyperlipidaemia								
* No*	1.075	0.984	1.173	-	1.144	1.000	1.308	-
* Yes*	1.596	0.861	2.960	0.127	0.700	0.229	2.136	0.505
COPD								
* No*	1.122	1.017	1.238	-	1.131	0.968	1.322	-
* Yes*	0.963	0.807	1.149	0.005	1.154	0.900	1.478	0.273
Cancer								
* No*	1.075	0.981	1.178	-	1.119	0.968	1.294	-
* Yes*	1.123	0.861	1.465	0.080	1.230	0.892	1.696	0.799

^a^Adjusted as Model 2

^**^p-values from Wald test

**Fig. 1:**
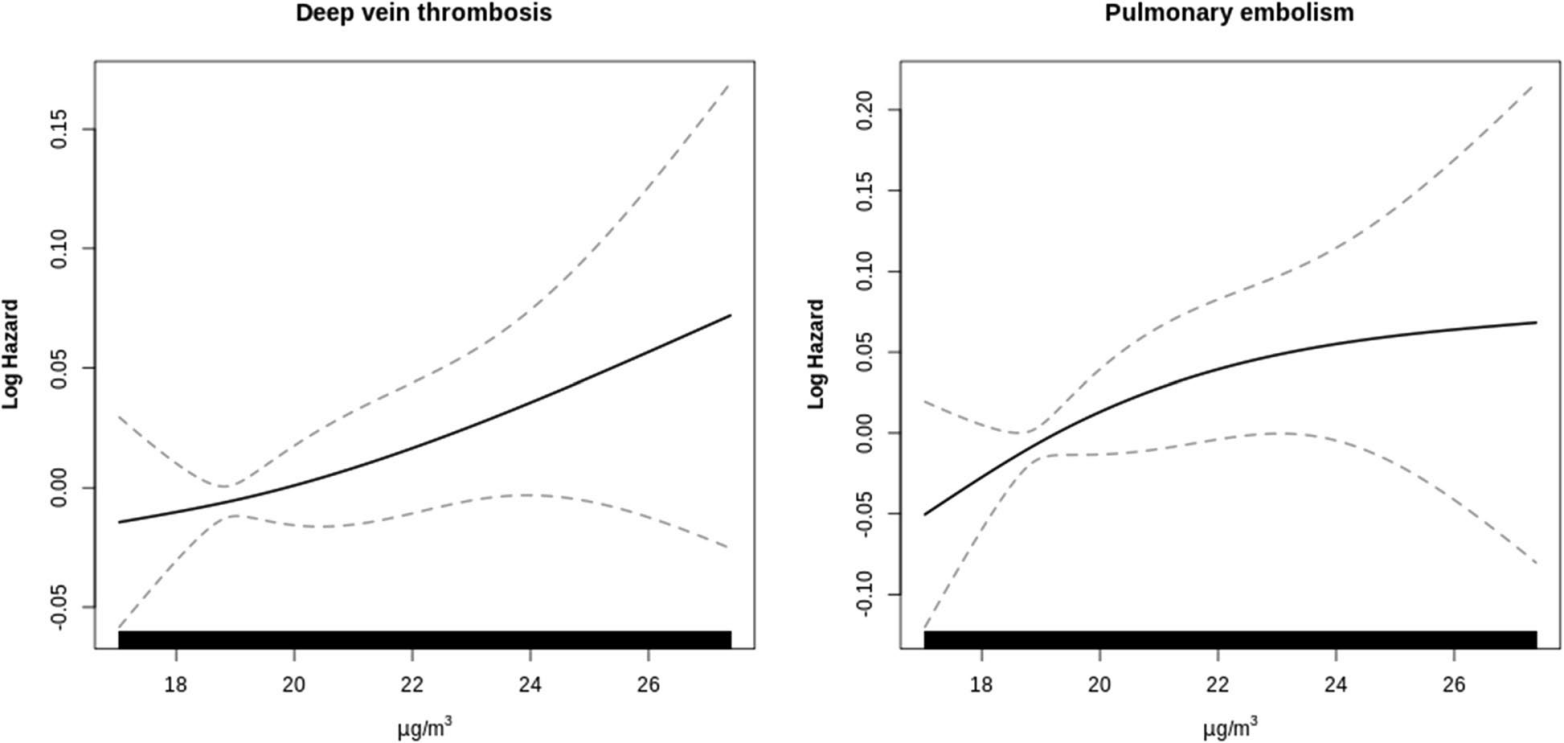
Exposure–response relationship between PM2.5 exposure and PE/DVT using Model 2 adjustment

Figure 1 reported the exposure–response curves for PM_2.5_ and DVT and PE. The pattern displayed by the curves was approximately linear for each pollutant.

In the Supplementary Table S3, we have reported the results of the sensitivity analysis considering the cluster term of spatial zones in Rome city. The estimates did not differ from those obtained in the main analyses.

## Discussion

We hypothesized that long-term exposure to air pollutants could increase the risk of hospitalization for DVT and PE. In this cohort study, we found an incidence of DVT of 9.8 per 10,000 person-years and an incidence of PE of 4.0 per 10,000 person-years which are lower compared to previously published estimates for US [9] and the EU [6]. In our main analysis as well as those adjusted for noise and greenness we found a 3–4% effect of PM_10_ and PM_2.5_ respectively on DVT, effects that are similar to those obtained in other CVD outcomes in studies with similar design to our [35]. The effect of pollutants on PE and VTE was higher although marginally non-significant, and should be interpreted as suggestive of a health effect that deserves attention in future studies.

Despite plausible biological mechanisms through the inflammatory and/or the autonomic imbalance pathways [36], epidemiological studies have reported contrasting results on the association between air-pollution exposure on VTE. A recent meta-analysis [20] found only three studies dealing with the long-term effect of air pollution (two cohort: [17, 18]; one case–control [37]:) and few other assessing the short-term effect [38–42]. All meta-analytic coefficients showed no differences from the null, although some of them were suggestive a detrimental effect of exposure [20]. Contrary to the above-mentioned studies, our analysis showed an increased risk of PE and DVT with all pollutants examined, possibly because of methodological differences between our and the other cohort studies. In fact, the study of Shih was restricted to 26,450 postmenopausal women 50–79 years of age while the study of Kan included 13,143 middle-aged men and women from the general population, using traffic density and distance to major roads as measures of traffic exposures and the Baccarelli study was case–control design. In our study, we had more statistical power to assess the effect as we followed through registries > 1 million of people while exposure was determined using standard LUR methodologies validated at the European level with a large collaborative project [26, 43]. Regarding the assessment of short-term effect of air pollution, it should be noticed that PE and DVT hospitalizations exhibit a marked seasonality that should be explicitly addressed in the study design as we found and effect of short-term exposure to PM_2.5_ on PE hospitalization in warmer months only [44].

In epidemiological literature there are not studies focused on the role of greenness and noise exposures on risk of VTE episodes. However, the protective effect of greenness on cardiovascular morbidity and mortality is well documented [45]. Analogously, noise exposure has been identified as an important risk factor for cardiovascular outcomes [46]. In our study, noise plays a clear negative confounding effect on the relationship between PM and outcomes, while no confounding was observed for NO_2_, probably due to the common source shared by them (vehicular traffic). On the other hand, greenness seems not to display an active role on these associations.

In effect modification analyses, we found slightly higher effects of PM on hospitalizations for DVT of younger subjects (< 70 years), and who had a cancer diagnosis and who had not COPD especially for DVT. This result is probably explained by the additional risk due to air pollution in individuals affected i.e. by COPD, while the result about cancer might by due by a more likely longer hospitalizations and relative bed immobilizations which occurred in those subjects.

Exposure–response relationship showed linear associations both for DVT and PE, as similar results were reported in previous studies on other cardiovascular outcomes (CIT). Moreover, risks were not positive at low concentrations. The absence of threshold and a linear relationship between exposure and DVT and PE hospitalization risk underlies the harmful effect of air pollutants also a low concentration, and below the European law limits [47]. From a public health perspective, general practitioners dealing with reduced mobility patients in the elderly and residing in areas of Rome with large pollution load should be conscious of the higher VTE risk for their patients and adjust their practice/preventive advice accordingly.

Some limitations of this study need to be mentioned. The studied cohort is based on administrative data (the Rome Longitudinal Study [21],) and not all individual risk factors for VTE were available such as smoking, BMI, physical activity, diet, hormone replacement therapies for women. However, we adjusted for socio-economic status that is known to be a predictor of unhealthy lifestyle [48] and for many CVD comorbidities that are also related to bad individual habits, therefore possibly accounting for part of the unmeasured risk factor effects. We are not able to detect all subjects treated at home for not critical DVT/PE episodes leading to a possible underestimation of the effect estimates. We assume that the spatial distribution of exposure did not change during the follow-up [49] from the time of LUR modelling setup. Also, as in many other similar studies, the individual exposure is attributed at the residential address and is therefore more precise for less mobile individuals such as the elderly. Furthermore, we excluded from the analysis subjects who moved from Rome during the study period or that did not have a known residential address. However, the sociodemographic characteristics of individual excluded were like those included and bias is unlikely [30, 50].

Among the strengths of this study, results are based on a large cohort of over 1 million of individuals followed for > 14y and overcome the possible low statistical power of previous longitudinal studies [17, 18], as confidence intervals of effect measures were larger than those measured in our study. Exposure was estimated at the residential addresses of the entire cohort, with some socio-demographic information individually available. Additionally, the available data on noise and greenness allowed for adjustment for those two important additional exposures that often confound the effect estimate of air pollutants on CVDs. Notably, effect estimates of air pollution adjusted for noise (lden) were higher, possibly because the measured air pollutants have only a partial share with road traffic emissions that, on the other hand, is the main source of noise in Rome [30].

In conclusion, we observed a positive association between long-term exposure to PM and risk of deep vein thrombosis and pulmonary embolism in a large administrative cohort in Rome. However, more statistical power is requested to infer properly on this study question. Future studies focused on the relationship between air pollution and pulmonary embolism or deep vein thrombosis are needed to explore deeply this investigation and give more precise indication to policy makers.

## Supplementary Information

**Additional file 1:** **Table S1.** Beta coefficients for Noise, Greenness and noise adjusted for greenness in Model 3 and 4 respectively. **Table S2.** Effect modification of the associations between long-term exposure to PM_2.5_ and deep vein.thrombosis and pulmonary embolism in RoLS for age category. Results are expressed as hazard ratios (HR) with relative 95% confidence intervals per 10 µg/m^3^ increases of pollutant. **Table S3.** Associations between long-term exposure to air pollutants (PM10, PM2.5 and NO2) and deep vein thrombosis (DVT) and pulmonary embolism (PE) in RoLS adjusted with a cluster term for the urban zone. Results are expressed as hazard ratios (HR) with relative 95% confidence intervals per 10 mg/m3 increases of pollutant.

## Data Availability

The data that support the findings of this study are available on request from the corresponding author [MR]. The data are not publicly available due to [privacy restriction: them containing information that could compromise research participant privacy/consent].
