# Supplementary material for: Long-term exposure to air pollution and risk of venous thromboembolism in a large administrative cohort
Source: Environ Health. 2022 Jan 27;21:21. doi: 10.1186/s12940-022-00834-2 (PMC8793234; doi:10.1186/s12940-022-00834-2)
Supplement: Supplementary file 1 — Additional file 1: Table S1. Beta coefficients for Noise, Greenness and noise adjusted for greenness in Model 3 and 4 respectively. Table S2. Effect modification of the associations between long-term exposure to PM2.5 and deep vein.thrombosis and pulmonary embolism in RoLS for age category. Results are expressed as hazard ratios (HR) with relative 95% confidence intervals per 10 µg/m3 increases of pollutant. Table S3. Associations between long-term exposure to air pollutants (PM10, PM2.5 and NO2) and deep vein thrombosis (DVT) and pulmonary embolism (PE) in RoLS adjusted with a cluster term for the urban zone. Results are expressed as hazard ratios (HR) with relative 95% confidence intervals per 10 mg/m3 increases of pollutant. [file 12940_2022_834_MOESM1_ESM.docx]

**Title:** Long-term exposure to air pollution and risk of venous thromboembolism in a large administrative cohort

**Authors:** Matteo Renzi^1,2^, Massimo Stafoggia^1,3^, Paola Michelozzi^1^, Marina Davoli^1^, Francesco Forastiere^4^ and Angelo G. Solimini^5^

**Affiliations:** 1) Department of Epidemiology, Health Authority Service, ASL Rome 1, Rome, Italy

2) Department of Health Statistics and Biometry, University of Rome “La Sapienza”, Rome, Italy

3) Institute of Environmental Medicine, Karolinska Instituet, Stockholm, Sweden

4) National Research Council of Italy, Institute of Innovation and Biomedical Research (IRIB), Palermo, Italy

5) Department of Public Health and Infectious Diseases, University of Rome “La Sapienza”, Rome, Italy

**Corresponding author:** Matteo Renzi, MSc

**Address:** Via Cristoforo Colombo 112, 00147, Rome, Italy

**E-mail:** m.renzi@deplazio.it

**Phone:** +39-0699722170

**Short running title:** Air pollution and venous thromboembolism

**Keywords:** air pollution, cohort, deep vein thrombosis, pulmonary embolism, venous thromboembolisms

**TABLE S1** Beta coefficients for Noise, Greenness and noise adjusted for greenness in Model 3 and 4 respectively.

|  | **PM_10_** | | **PM_2.5_** | | **NO_2_** | |
| --- | --- | --- | --- | --- | --- | --- |
|  | **log(beta)** | **se** | **beta** | **se** | **beta** | **se** |
| **DVT** |  |  |  |  |  |  |
|  |  |  |  |  |  |  |
| *Noise* | -0.00067 | 0.00116 | -0.00073 | 0.00117 | 0.00076 | 0.00118 |
| *Greenness* | -0.15168 | 0.08930 | -0.14263 | 0.08968 | -0.14264 | 0.09363 |
| *Noise adj for greenness* | 0.00013 | 0.00189 | 0.00087 | 0.00189 | 0.00067 | 0.00167 |
|  |  |  |  |  |  |  |
| **PE** |  |  |  |  |  |  |
|  |  |  |  |  |  |  |
| *Noise* | 0.00072 | 0.00186 | 0.00053 | 0.00187 | 0.00104 | 0.00188 |
| *Greenness* | -0.15168 | 0.08929 | -0.14263 | 0.08968 | -0.14264 | 0.09363 |
| *Noise adj for greenness* | 0.00013 | 0.00189 | 0.00087 | 0.00189 | 0.00091 | 0.00188 |
|  |  |  |  |  |  |  |
| **VTE** |  |  |  |  |  |  |
|  |  |  |  |  |  |  |
| *Noise* | -0.00044 | 0.00101 | -0.00051 | 0.00102 | 0.00062 | 0.00103 |
| *Greenness* | 0.07852 | 0.04871 | 0.08669 | 0.04895 | 0.07763 | 0.05118 |
| *Noise adj for greenness* | -0.00012 | 0.00103 | -0.00023 | 0.00103 | 0.00070 | 0.00103 |

|  |  | **DVT** | | |  | **PE** | | |  |
| --- | --- | --- | --- | --- | --- | --- | --- | --- | --- |
|  |  | HR | 95%*CI | | p-value^**^ | HR | 95%CI | | p-value |
|  |  |  |  |  |  |  |  |  |  |
|  |  |  |  |  |  |  |  |  |  |
| Age class | *<40 y* | 0.967 | 0.714 | 1.311 | - | 1.356 | 0.728 | 2.525 | - |
|  | *>40 & <65 y* | 1.109 | 0.967 | 1.271 | - | 1.289 | 1.032 | 1.609 | - |
|  | *>65 y* | 1.081 | 0.959 | 1.219 | 0.502 | 1.097 | 0.925 | 1.304 | 0.520 |

**TABLE S2** Effect modification of the associations between long-term exposure to PM_2.5_ and deep vein thrombosis and pulmonary embolism in RoLS for age category. Results are expressed as hazard ratios (HR) with relative 95% confidence intervals per 10 μg/m^3^ increases of pollutant

| **Pollutant** | **beta** | **se** | **AIC** | **BIC** | **HR** | **95%low** | **95%up** | **model** | **outcome** |
| --- | --- | --- | --- | --- | --- | --- | --- | --- | --- |
| PM10 | 0.00377 | 0.00244 | 136499 | 136505 | 1.038 | 0.990 | 1.089 | 1 | PE |
| PM2.5 | 0.01048 | 0.00661 | 136499 | 136505 | 1.110 | 0.976 | 1.264 | 1 | PE |
| NO2 | 0.00163 | 0.00127 | 136499 | 136506 | 1.016 | 0.992 | 1.042 | 1 | PE |
| PM10 | 0.00426 | 0.00249 | 136269 | 136476 | 1.044 | 0.994 | 1.096 | 2 | PE |
| PM2.5 | 0.01272 | 0.00681 | 136268 | 136475 | 1.136 | 0.994 | 1.298 | 2 | PE |
| NO2 | 0.00188 | 0.00137 | 136270 | 136477 | 1.019 | 0.992 | 1.047 | 2 | PE |
| PM10 | 0.00371 | 0.00286 | 136270 | 136484 | 1.038 | 0.981 | 1.098 | 3 | PE |
| PM2.5 | 0.01159 | 0.00788 | 136270 | 136484 | 1.123 | 0.962 | 1.310 | 3 | PE |
| NO2 | 0.00144 | 0.00159 | 136271 | 136485 | 1.014 | 0.983 | 1.047 | 3 | PE |
| PM10 | 0.00362 | 0.00286 | 136270 | 136490 | 1.037 | 0.980 | 1.097 | 4 | PE |
| PM2.5 | 0.01038 | 0.00792 | 136269 | 136490 | 1.109 | 0.950 | 1.296 | 4 | PE |
| NO2 | 0.00067 | 0.00167 | 136271 | 136492 | 1.007 | 0.974 | 1.040 | 4 | PE |
| PM10 | 3E-06 | 0.00159 | 341852 | 341860 | 1.000 | 0.969 | 1.032 | 1 | DVT |
| PM2.5 | -0.00077 | 0.00432 | 341852 | 341860 | 0.992 | 0.912 | 1.080 | 1 | DVT |
| NO2 | -0.00027 | 0.00081 | 341852 | 341860 | 0.997 | 0.982 | 1.013 | 1 | DVT |
| PM10 | 0.00283 | 0.00162 | 340583 | 340818 | 1.029 | 0.996 | 1.062 | 2 | DVT |
| PM2.5 | 0.00789 | 0.00444 | 340583 | 340818 | 1.082 | 0.992 | 1.181 | 2 | DVT |
| NO2 | -0.00043 | 0.00088 | 340586 | 340821 | 0.996 | 0.979 | 1.013 | 2 | DVT |
| PM10 | 0.00334 | 0.00185 | 340584 | 340828 | 1.034 | 0.997 | 1.072 | 3 | DVT |
| PM2.5 | 0.00945 | 0.00509 | 340584 | 340827 | 1.099 | 0.995 | 1.214 | 3 | DVT |
| NO2 | -0.00076 | 0.00101 | 340587 | 340830 | 0.992 | 0.973 | 1.012 | 3 | DVT |
| PM10 | 0.00345 | 0.00185 | 340580 | 340831 | 1.035 | 0.998 | 1.073 | 4 | DVT |
| PM2.5 | 0.0109 | 0.00511 | 340579 | 340830 | 1.115 | 1.009 | 1.233 | 4 | DVT |
| NO2 | 3E-05 | 0.00106 | 340583 | 340834 | 1.000 | 0.980 | 1.021 | 4 | DVT |

**Table S3**. Associations between long-term exposure to air pollutants (PM10, PM2.5 and NO2) and deep vein thrombosis (DVT) and pulmonary embolism (PE) in RoLS adjusted with a cluster term for the urban zone. Results are expressed as hazard ratios (HR) with relative 95% confidence intervals per 10 μg/m3 increases of pollutant.
